# Knowledge mapping and research trends of accidental falls in patients with Parkinson’s disease from 2003 to 2023: a bibliometric analysis

**DOI:** 10.3389/fneur.2024.1443799

**Published:** 2024-08-22

**Authors:** Luya Shi, Bongsook Yih

**Affiliations:** ^1^Municipal Hospital Affiliated to Taizhou University, Taizhou, Zhejiang, China; ^2^Department of Graduate, School of Nursing, Sehan University, Yeonggam, Republic of Korea

**Keywords:** accidental falls, bibliometric, CiteSpace, Parkinson’s disease, VOSviewer

## Abstract

**Background:**

Recent years have witnessed a rapid growth in research on accidental falls in patients with Parkinson’s Disease (PD). However, a comprehensive and systematic bibliometric analysis is still lacking. This study aims to systematically analyze the current status and development trends of research related to accidental falls in patients with PD using bibliometric methods.

**Methods:**

We retrieved literature related to accidental falls in patients with PD published between January 1, 2003, and December 31, 2023, from the Web of Science Core Collection (WoSCC) database. Statistical analysis and knowledge mapping of the literature were conducted using VOSviewer, CiteSpace, and Microsoft Excel software.

**Results:**

A total of 3,195 publications related to accidental falls in patients with PD were retrieved. These articles were authored by 13,202 researchers from 3,834 institutions across 87 countries and published in 200 academic journals. Over the past 20 years, the number of published articles and citations has increased annually. The United States and the United Kingdom have the highest number of publications in this field, while Harvard University and Tel Aviv University are the most influential institutions. The *Parkinsonism & Related Disorders* journal published the highest number of articles, while the *Movement Disorders* journal had the highest number of citations. The most prolific author is Bloem, Bastiaan R, while the most cited author is Hausdorff, Jeffrey. The main research areas of these publications are Neurosciences, Biomedical, Electrical & Electronic, and Biochemistry & Molecular Biology. Currently, high-frequency keywords related to accidental falls in patients with PD include risk factors, clinical manifestations, and interventions. Prediction and prevention of accidental falls in such patients is a research topic with significant potential and is currently a major focus of research.

**Conclusion:**

This study used bibliometric and knowledge mapping analysis to reveal the current research status and hotspots in the field of accidental falls in patients with PD. It also points out directions for future research. This study can provide theoretical support and practical guidance for scholars to further conduct related research.

## 1 Introduction

Falls in patients with Parkinson’s Disease (PD) are a common yet serious issue, with 45–68% of patients with PD worldwide experiencing falls annually, two-thirds of whom experience recurrent falls. This rate is twice that of falls in non-PD older adults making it the second leading cause of accidental death in the older population (1–3). Falls not only cause physiological pain such as fractures and injuries, but also lead to a fear of falling, reduced mobility, and a series of negative impacts, which in turn exacerbate disease symptoms, reduce the quality of life, and increase medical costs (4, 5). Therefore, preventing and controlling falls is crucial for improving the quality of life of patients with PD, delaying disease progression, and reducing the burden on society and families (1).

Falls in patients with PD are associated with various factors, such as impaired balance, motor function deficits, freezing of gait (FoG), and postural instability (6–8). Although multiple studies have reported on attempts to prevent and reduce falls in these patients, there is still a lack of effective preventive measures (2, 9). Recent studies are increasing their focus on identifying risk factors for falls in patients with PD, understanding the pathological mechanisms, clinical characteristics of falls, and developing preventive and intervention measures. However, the research directions and hotspots in this field still remain unclear (10). Therefore, it is necessary to conduct a bibliometric analysis of the research on falls in patients with PD to gain a comprehensive understanding of the current state and research hotspots in this field and to clarify future research directions.

Existing bibliometric analyses have reported scientific achievements related to deep brain stimulation, anxiety and depression, non-motor symptoms, and prodromal symptoms of PD as temporal trends, geographical distribution, and influencing factors. However, to our knowledge, no bibliometric analysis focusing on falls in patients with PD has yet been conducted. Bibliometric analysis employs mathematical and statistical techniques to conduct both qualitative and quantitative assessments of research within a specific field over a designated period (11). This method emphasizes examining countries, institutions, journals, authors, and keywords associated with the field of study, providing readers with an objective view of trends and emerging frontiers in that area (12). Bibliometric analysis has been extensively utilized across various fields, including oncology, cardiovascular diseases, endocrinology, and traditional Chinese medicine, which has helped in obtaining clear insights into related research (13–16). However, the knowledge structure, evolution paths, and research hotspots concerning falls in patients with PD have not yet been analyzed from a bibliometric perspective. As significant progress has been made in research related to falls in such patients over the past 2 decades, it is necessary, and timely, to conduct a bibliometric study in this field to provide researchers with intuitive visual information and potential research directions. This study aims to utilize VOSviewer and CiteSpace, two bibliometric software tools, to comprehensively analyze research on falls in patients with PD published over the past 20 years. This analysis will construct a knowledge map of the field, identify and analyze development trends and research hotspots, provide researchers with valuable references, and guide further in-depth studies in this area.

## 2 Materials and methods

### 2.1 Data collection

We collected relevant data from the Web of Science Core Collection (WoSCC) database, one of the largest and most authoritative electronic scientific literature databases in the world (17). We retrieved literature published between January 1, 2003, and December 31, 2023. To avoid errors, we completed the download from the WoSCC database in a single session on May 25, 2024. The search terms used in this study were as follows: “Accidental Fall*” OR “Fall* Accidental” OR “fall*” OR “Slip and Fall” OR “Fall and Slip” AND “Parkinson Disease” OR “Parkinson*” OR “PD.” The document types were limited to Article or Review Article, excluding retracted publications. The search results were exported in “plain text” and “full record and cited references” formats. Ultimately, 3,195 original articles and review articles were included.

### 2.2 Data analysis

We imported the downloaded data into CiteSpace 6.3.R1, VOSviewer 1.6.20 and Microsoft Excel 2019 for bibliometric and knowledge mapping analyses. To ensure data accuracy, synonyms were merged, and different spellings of author names and institution names were standardized before conducting the keyword co-occurrence analysis.

General information about the literature was extracted from Web of Science (WOS) and organized using Microsoft Excel 2019.

VOSviewer, developed by Nees Jan van Eck and Ludo Waltman, is a bibliometric analysis software tool that constructs and visualizes bibliometric knowledge maps based on network data, extracting key information from numerous publications (18). It can create visual network maps based on collaboration data and generate keyword knowledge maps based on co-occurrence information. The main goal of VOSviewer is to provide users with a comprehensive view of the dynamic development and internal structure of scientific research.

CiteSpace, developed by Professor Chaomei Chen, is a Java-based bibliometric and visualization analysis platform (19). It can visualize the contributions and collaboration relationships of countries and institutions and analyze disciplinary distribution, citations, co-citations and research hotspots, thereby uncovering potential information and patterns within large volumes of literature data.

## 3 Results

### 3.1 Annual publication growth trend

**Figure 1:**
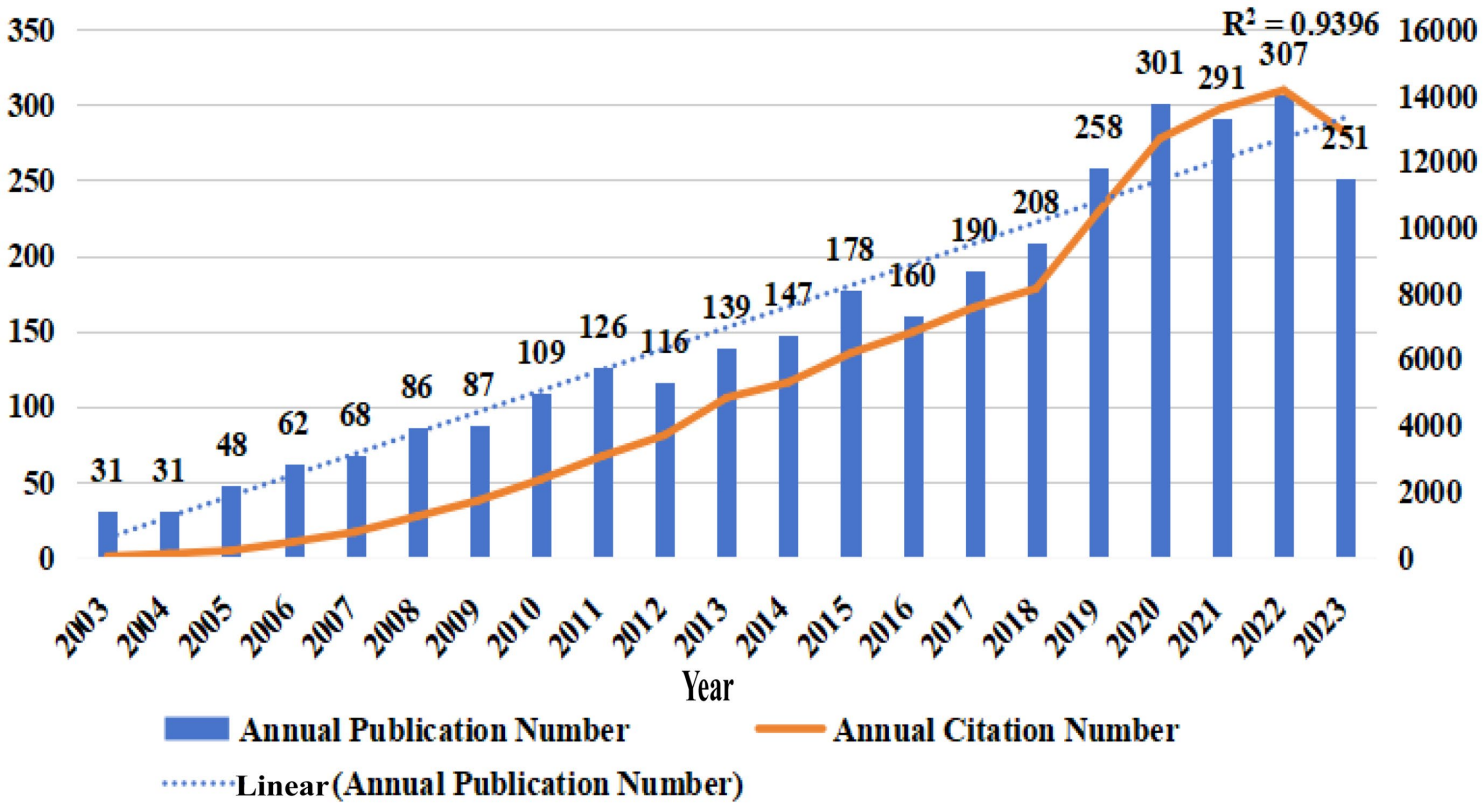
Temporal trends in annual publications and citations related to accidental falls among individuals with Parkinson’s Disease (PD) from 2003 to 2023. The number of publications (bar) and citations (solid line) have exhibited a steady upward trajectory, reflecting a growing research interest and emphasis on this critical issue within the PD population.

The yearly publication trends in a specific research area serve as an intuitive indicator of the global involvement and level of interest in that field. It is a simple yet insightful analytical method. Our research strategy helped retrieve 3,195 publications related to accidental falls in patients with PD from the WoSCC database. As shown in Figure 1, the annual publication and citation volumes in the field of “accidental falls in patients with PD” have shown a steady upward trend from 2003 to 2023. The number of publications increased from only 31 in 2003 and 2004 to 307 in 2022, with a high correlation coefficient (*R*^2^ = 0.9396) indicating a strong linear growth trend. During the same period, the annual citation volume of literature in this field also showed rapid growth.

The continual increase in both the number of publications (*N_p_*) and citations (*N_c_*) suggests a growing interest and significant scholarly investment in the study of accidental falls among patients with PD. The issue of falls, a common complication in the patient population with PD, has garnered increasing attention from the scientific community and has become a hot research topic. Hence, there is an urgent need in clinical practice to prevent and manage the risk of falls in this patient group, which aligns with the modern medical focus on improving the quality of life of patients.

### 3.2 Analysis of countries and institutions

**Figure 2:**
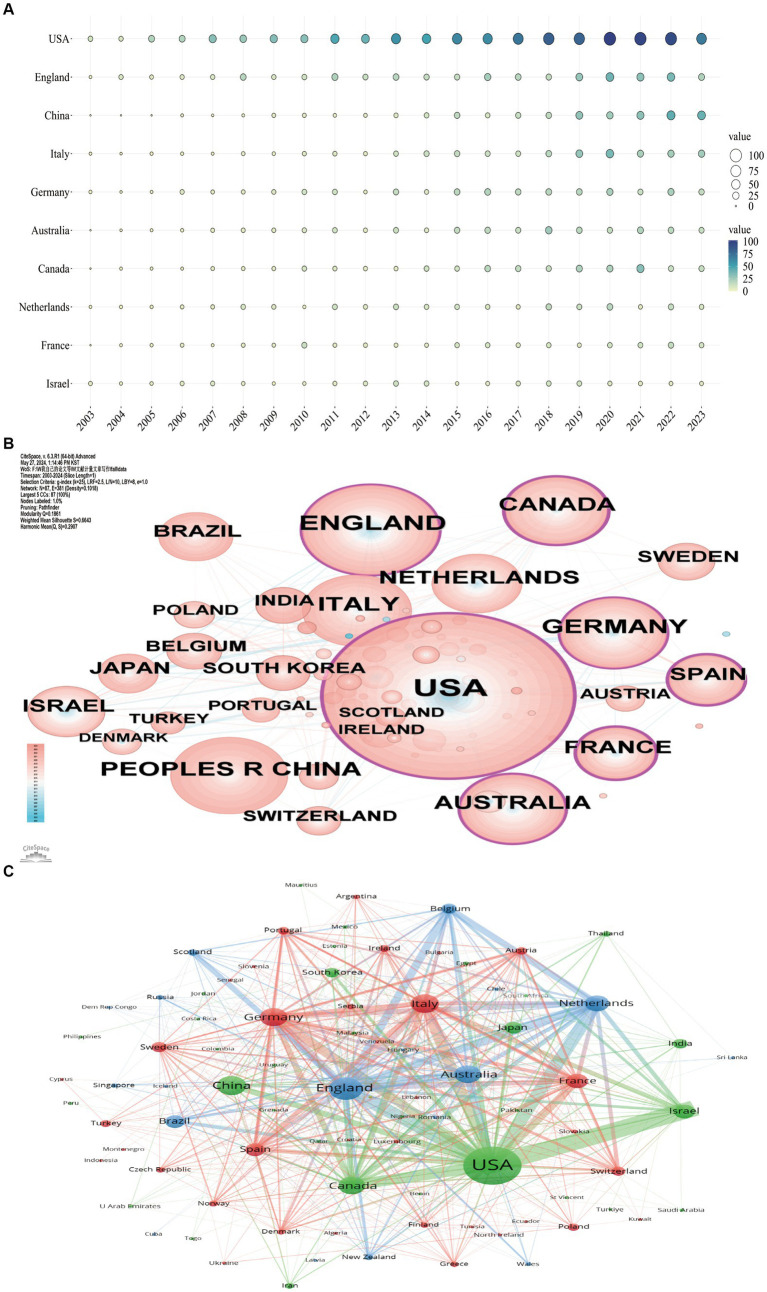
Geospatial analysis of research output related to accidental falls in individuals with Parkinson’s Disease (PD). **(A)** Temporal trends in annual publication output from the top 10 prolific countries. Node size represents the annual publication count. **(B)** Co-occurrence network of contributing countries. Here, node size corresponds to the frequency of co-occurrence, while nodes encircled in purple indicate high betweenness centrality (≥0.1), indicating influential bridging roles. **(C)** Collaboration network among countries. Node size reflects publication frequency, and links represent co-authorship relationships. Node colors denote distinct country clusters based on collaborative patterns.

National collaboration networks help in identifying the most influential countries in the field. In the present study, 87 countries and 3,834 institutions participated in research related to accidental falls in patients with PD. Based on indicators such as *N_p_*, *N_c_*, H-index, and total link strength (TLS), we ranked the top 10 countries with the highest publication volumes (Supplementary Table S1). Figure 2A shows the annual publication volumes of these 10 countries from 2003 to 2023. In terms of publication volume, the United States leads the list with 1,117 papers, which is approximately three times that of the second-ranked United Kingdom (377 papers). China was in the third place with 282 papers. In terms of total citations, the United States again leads significantly with 53,611 citations, followed by the United Kingdom (20,064 citations) and Australia (16,949 citations). The H-index also places the United States (111), the United Kingdom (70), and the Netherlands (62) in the top 3. Hence, it can be suggested that in terms of the quality, quantity, and impact of papers, the United States and the United Kingdom far surpass other countries and dominate the field.

Additionally, there are close cooperative relationships between countries, as shown in Figures 2B,C. In Figure 2B, countries with purple circular nodes represent high betweenness centrality (≥0.1), with the top 5 being the United States (0.34), Canada (0.24), Spain (0.24), France (0.22), and the United Kingdom (0.20), reflecting their crucial roles in the research on “accidental falls in PD patients.” Figure 2C illustrates the national collaboration network, which shows that the United States, Israel, the Netherlands, England, and Canada, which are among the top 10 publishing countries, have close cooperation, with the USA and Israel having the closest collaboration.

**Figure 3:**
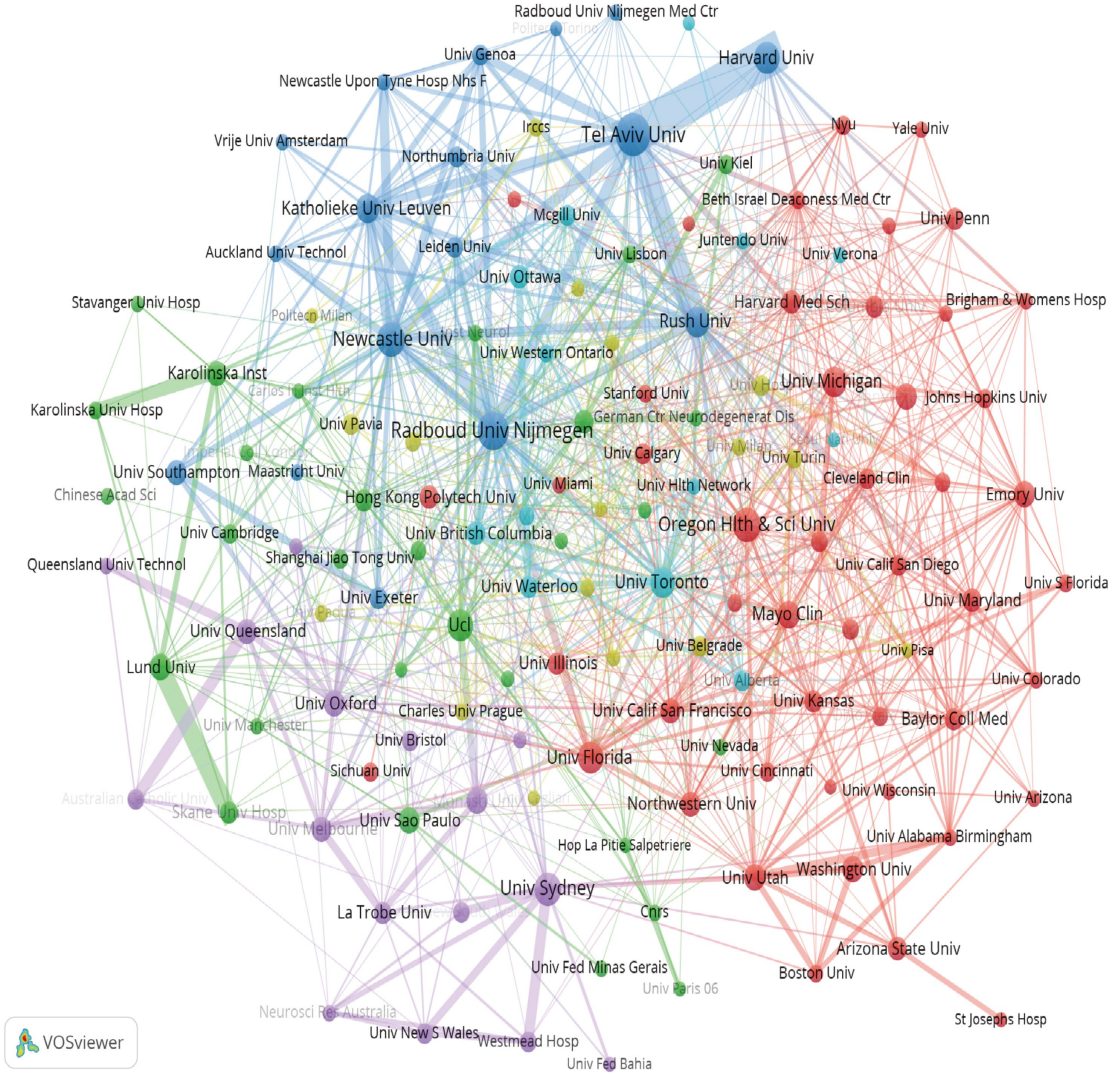
Institutional collaboration network in research on accidental falls among individuals with Parkinson’s Disease (PD). Node colors denote distinct institutional clusters based on collaborative patterns. Node size reflects the total publication output of each institution. Link width is proportional to the strength of inter-institutional collaboration.

Among the top 10 research institutions in terms of publication volume and citation frequency, Tel Aviv University of Israel ranks first in term of publication volume (119), followed by the University of London of the United Kingdom (115) and Radboud University of the Netherlands (110) (Supplementary Table S2). In terms of *N_c_* and H-index, Harvard University and Tel Aviv University are the top 2, which reflects the strength and impact of their research. Based on the VOS visualization of institutional collaboration networks (occurrence frequency ≥ 10), a total of 140 institutions were grouped into 10 clusters. The largest cluster (blue) in Figure 3 consists of Tel Aviv University, Harvard University, and Rush University, indicating their close cooperation and significant role in the research on accidental falls in patients with PD.

### 3.3 Analysis of journals

Visual analysis of published journals and co-cited journals helps identify the influential core journals in the field. We identified 3,195 articles related to accidental falls in patients with PD across 200 academic journals. As shown in Supplementary Table S3, the top three journals with the highest number of publications are *Parkinsonism & Related Disorders* (143 articles, 4.48%), *Movement Disorders* (138 articles, 4.32%), and *Gait & Posture* (93 articles, 2.91%). These aforementioned three journals belong to the Q1 Journal Citation Reports (JCR) category. In terms of citations, *Movement Disorders* (15,766) ranks first, with approximately three times more citations than that in the second-ranked *Parkinsonism & Related Disorders* (5,313). Additionally, *Movement Disorders* (64) and *Parkinsonism & Related Disorders* (43) also rank first and second regarding the H-index, respectively.

**Figure 4:**
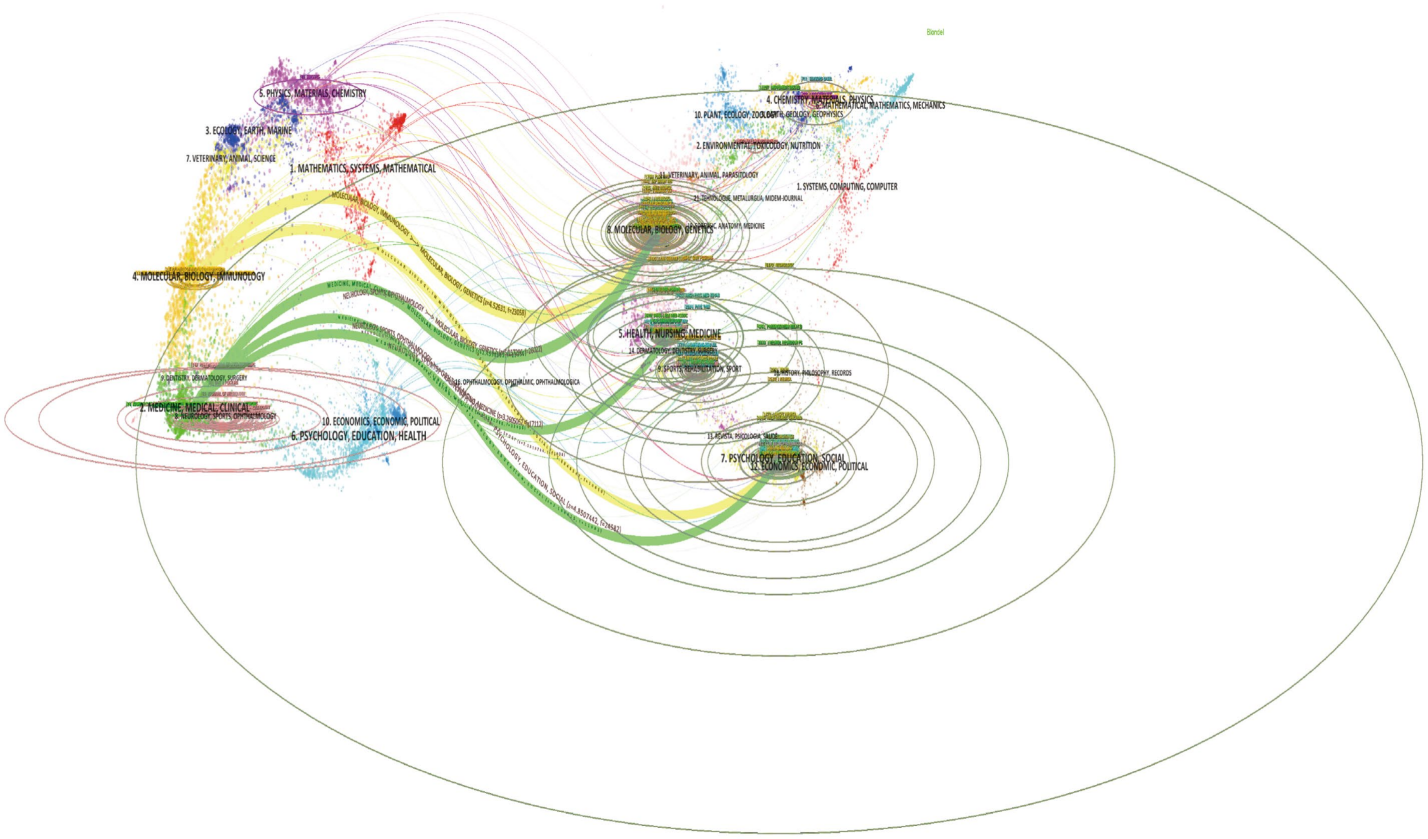
Dual-map overlay visualizing the citation landscape in research on accidental falls among individuals with Parkinson’s Disease (PD). On the left, clusters represent groups of citing journals that frequently reference literature within this domain. On the right, clusters depict the most frequently cited journals contributing seminal works. Colored trails connecting the two maps illustrate the citation relationships between the citing and cited journal clusters.

The overlay visualization of journals provides a more intuitive representation of the thematic distribution across individual journals, shifts in citation trajectories, and the evolution of research hubs. We employed knowledge flow analysis to explore the evolutionary patterns of knowledge citations and co-citation relationships among the cited journals. In the dual-map overlay, the labels on the left denote the citing journals, while those on the right represent the cited journals. The colorful curves depict citation links emanating from the citing map to the cited map, reflecting the contextual nature of the citations. Overall, as illustrated in Figure 4, the citing references predominantly originate from the fields of Molecular, Biology, Immunology/Medicine, Medical, and Clinical studies, which are generally considered research frontiers. Conversely, the cited references primarily belong to the domains of Molecular, Biology, Genetics/Health, Nursing, Medicine/Psychology, Education, and Social Sciences and are regarded as the knowledge base. Our analysis reveals the interdisciplinary knowledge flow and cross-fusion patterns in the field of accidental falls in patients with PD, reflecting the diversity of research in this area.

### 3.4 Analysis of authors

**Figure 5:**
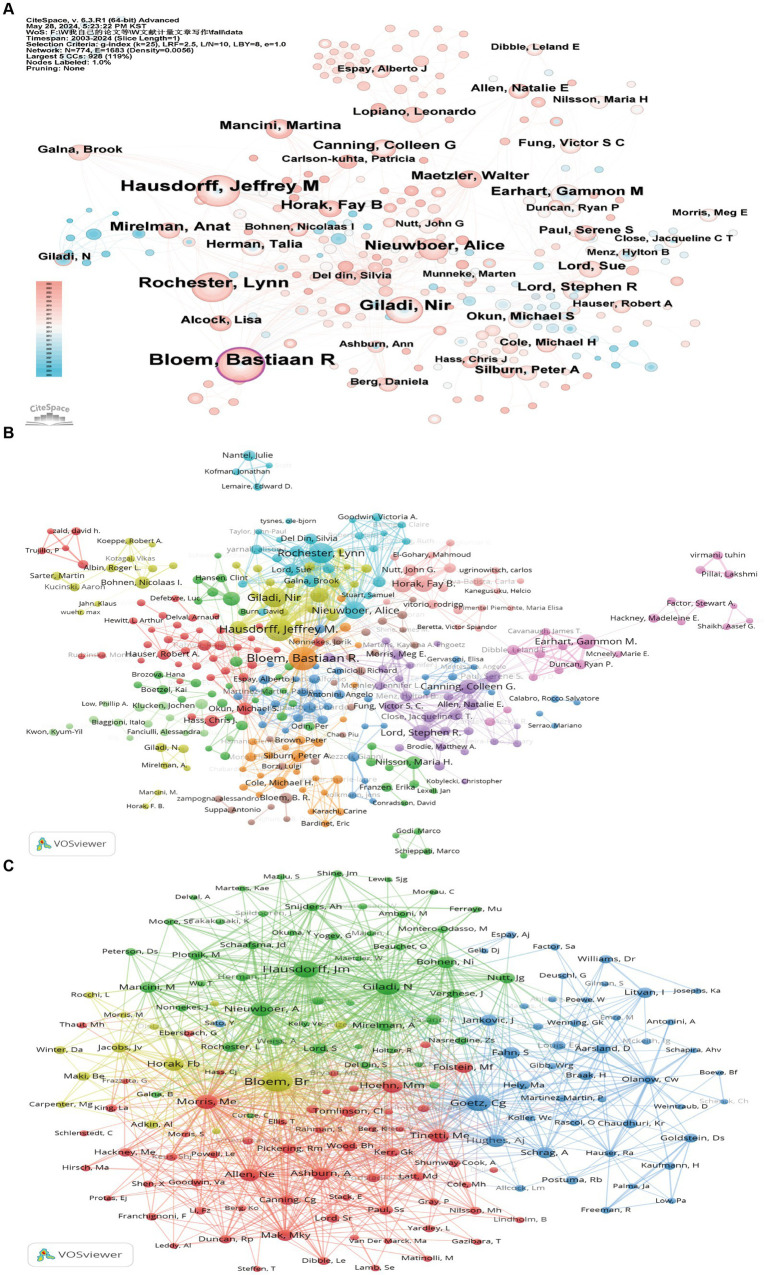
Analysis of authors in research on accidental falls among individuals with Parkinson’s Disease (PD). **(A)** Betweenness centrality of authors. Nodes encircled in purple indicate high betweenness centrality, indicating influential bridging roles connecting distinct author clusters. **(B)** Co-authorship network of contributing authors. Nodes are colored by cluster membership based on collaborative patterns. Node size corresponds to the frequency of co-authorship, while links represent co-authorship relationships between authors. **(C)** Co-citation network of influential authors. Node size reflects the frequency at which an author’s works are cited by others in this research domain. Nodes are colored by cluster membership based on collaborative patterns.

Author analysis helps identify the most influential researchers in the field. We considered 13,202 authors with published studies on accidental falls in patients with PD. Supplementary Table S4 lists the top 10 authors with the highest number of publications and citations. In terms of publications, Bloem, Bastiaan R from the Netherlands leads with 93 articles, followed closely by Hausdorff, Jeffrey (86) and Giladi, Nir (63), both from Israel. Regarding the total number of citations, Hausdorff, Jeffrey (12,021), Giladi, Nir (9,983), and Bloem, Bastiaan (7,477) are the top three researchers. These three scholars also top the list in terms of the H-index. Bloem, Bastiaan R has a high centrality (0.12), as shown in Figure 5A, indicating his significant influence within the research domain.

Figure 5B displays the author collaboration network, providing information on potential research partners. The figure includes 11 different colors representing 11 clusters. The largest cluster is centered around Bloem, Bastiaan R from the Netherlands (orange), the second-largest cluster (yellow) is centered around Hausdorff, Jeffrey from Israel, and the third-largest cluster (blue) converges around Rochester, Lynn from England, indicating close collaboration among them.

Author co-citation analysis reflects the similarity and strength of different authors in academic research. A high co-citation frequency between two authors indicates that their research fields are closely related. In Figure 5C 168 authors with more than 80 citations each, form four clusters centered around Bloem, Bastiaan R and Horak, Fay B (yellow); Hausdorff, Jeffrey and Giladi, Nir (green); Goetz CG and Hughes AJ (blue) and Morris ME and Ashburn A (red).

### 3.5 Analysis of reference

Highly cited literature can be regarded as the knowledge foundation of subfields, representing the key carriers of research field development. Supplementary Table S5 lists the top 10 most cited articles in the field of accidental falls in patients with PD. The table shows that the first six articles have over 1,000 citations, four of which are original research papers. The most-cited paper is “The Sydney multicenter study of Parkinson’s disease: The inevitability of dementia at 20 years” by Hely, Mariese A published in *Movement Disorders*, which systematically observed and followed up patients with PD and revealed that most patients experience varying degrees of severe non-motor symptoms, especially dementia, falls, and hallucinations.

**Figure 6:**
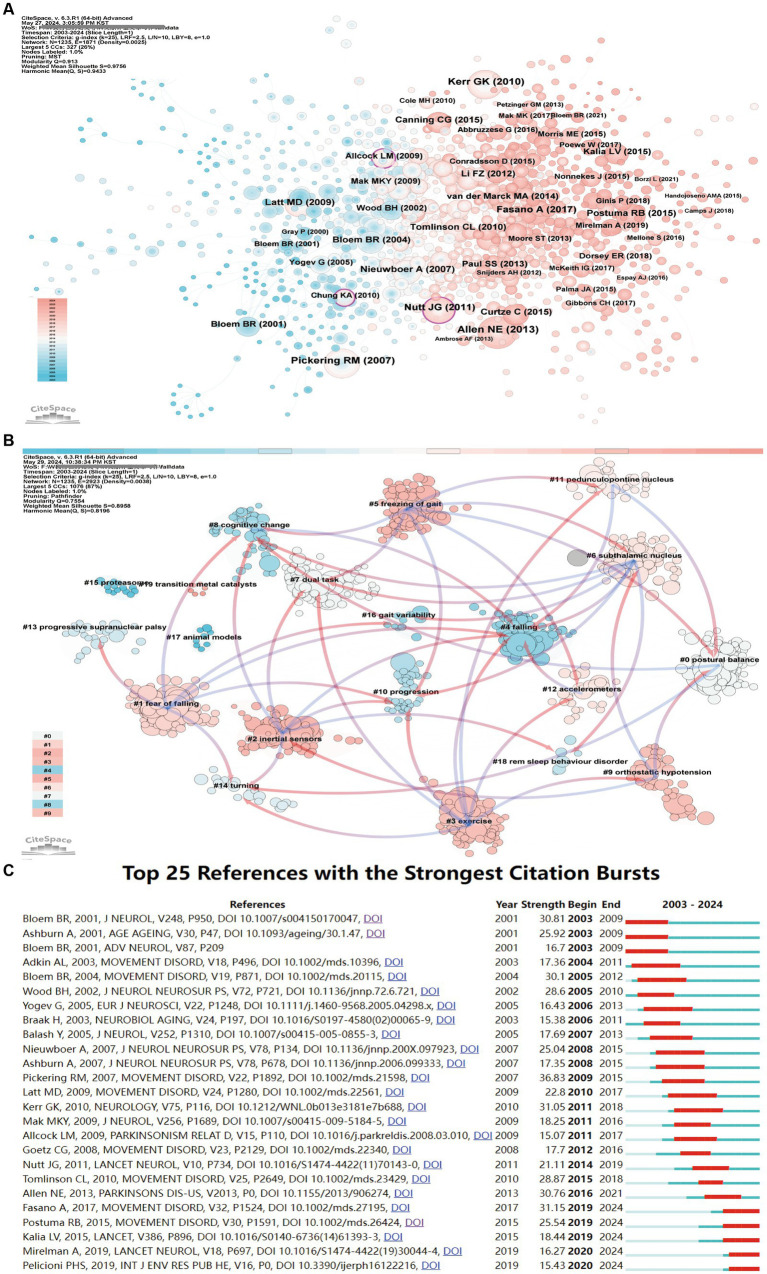
Reference analysis in research on accidental falls among individuals with Parkinson’s Disease (PD). **(A)** Co-citation network visualization. Node size represents citation frequency, while purple nodes indicate high betweenness centrality (≥0.1), suggesting influential works bridging distinct research clusters. Node color gradients from pink to blue depict temporal patterns, with pink indicating more recent publications. **(B)** Clustering of references based on semantic similarity, including #0 dual task, #1 fear of falling, #2 exercise, #3 balance impairment, #4 freezing of gait, #5 dance, #6 wearable technology, #7 festination, and others. Pink arrows indicate the primary citation flow within each cluster. **(C)** Top 25 references with the strongest citation bursts. Red bars indicate years with significantly elevated citation counts, revealing emergent publications that attracted intense scholarly attention over a specific period.

Utilization of co-citation cluster analysis can allow an objective demonstration of the knowledge structure of the research field (Figure 6A). “Freezing of gait: moving forward on a mysterious clinical phenomenon” by Nutt JG, published in *Lancet Neurology* in 2011, has the highest centrality, as it first proposed FoG as a feature of Parkinson’s syndrome, closely related to falls in patients with PD.

By visualizing the spread trajectory and residence time of co-cited footprints among different clusters, we can trace the evolution of knowledge in this field as well as the accumulation and exchange of information among all researchers. Based on the relevance of paper, we can place them in 23 clusters (Figure 6B). The largest cluster is postural balance (#0). Its study began with the use of animal models (#17) and gradually evolved into dual task (#7), cognitive change (#8), progression (#10), gait variability (#16), subthalamic nucleus (#6), and new hotspots such as fear of falling (#1), inertial sensors (#2), exercise (#3), and freezing of gait (#5). From the figure, it can be seen that postural balance (#0) evolved from cognitive change (#8) and turning (#14). It further evolved into freezing of gait (#5) and orthostatic hypotension (#9). Hence, it can be suggested that postural balance is the foundation of this research field. Inertial sensors (#2), exercise (#3), fear of falling (#1), and freezing of gait (#5) did not evolve into new clusters from other groups, indicating that they are current research hotspots as well as future research trends. Recent years have seen an increase in the density of connections between research fields.

Citation burst references refer to references that experience a significant and sudden increase in their citations within a certain period. Figure 6C lists the top 25 articles with the strongest citation bursts. The earliest citation burst began in 2001. The strongest burst (strength 36.83) was noted for the paper titled “A meta-analysis of six prospective studies of falling in Parkinson’s disease” by Pickering et al., which was published in *Movement Disorder*. This paper saw an explosive growth in citations from 2009 to 2015. The paper “Predictors of future falls in Parkinson disease” by Kerr et al., published in *Neurology* in 2010, has the longest burst cycle of 7 years (2011–2018). Five papers (two reviews and three articles), including “Gait impairments in Parkinson’s disease” by Mirelman Anat published in *Lancet Neurology* in 2019 and “Parkinson’s disease” by Kalia published in *Lancet Journal* in 2015, are currently experiencing bursts. They mainly focused on identifying clinical diagnostic criteria for motor disorders, gait impairment, subtypes of falls, identification assessment, and intervention for falls in PD, indicating that these are research hotspots and future trends in the field.

### 3.6 Analysis of hotspots and frontiers

**Figure 7:**
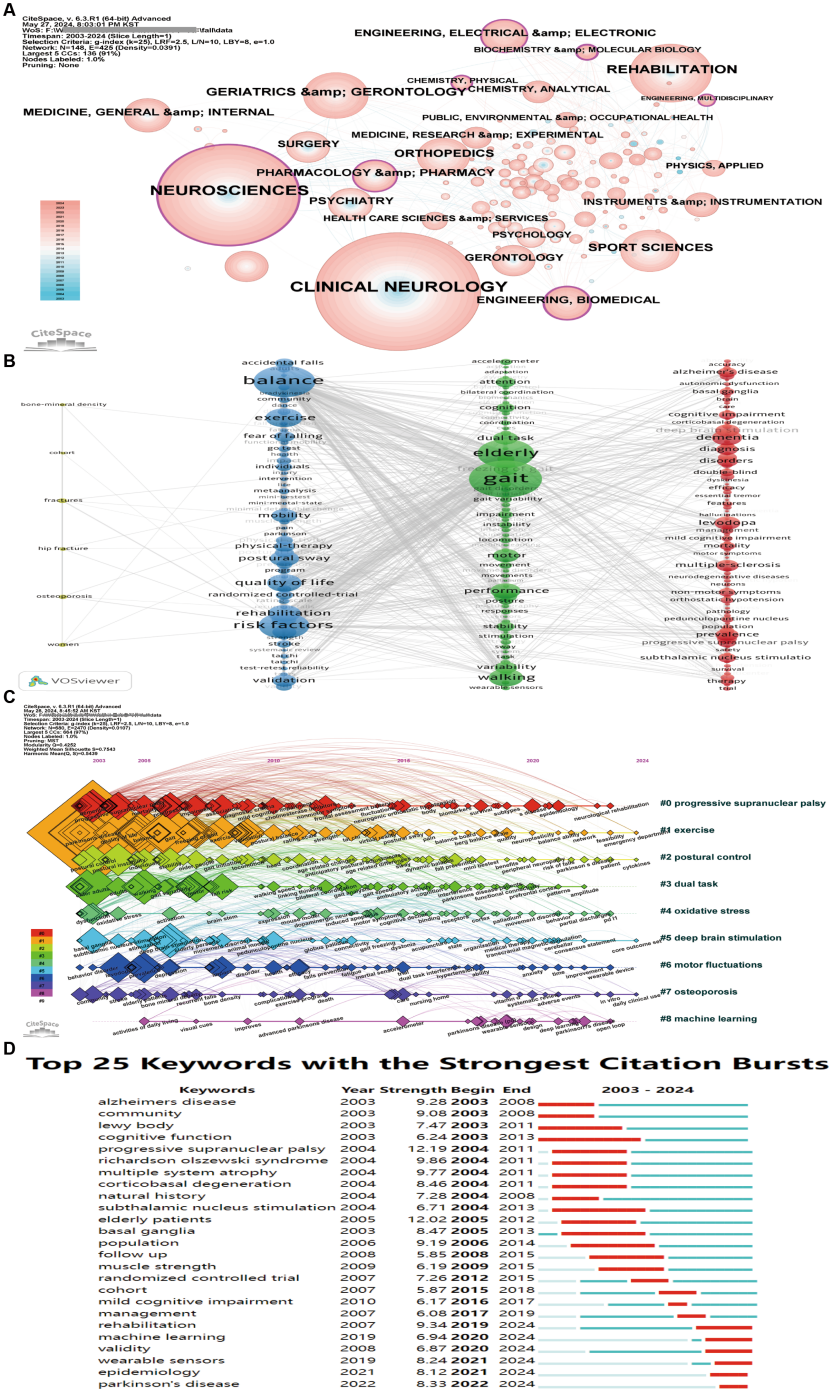
Analytical overview of research domains and prominent keywords in the study of accidental falls among individuals with Parkinson’s Disease (PD). **(A)** Co-occurrence network of contributing research fields/disciplines. Node size represents citation frequency, while purple nodes indicate high betweenness centrality (≥0.1), suggesting influential works bridging distinct research clusters. **(B)** Co-occurrence network of prominent keywords, where node size represents frequency and links indicate co-occurrence relationships. **(C)** Temporal view depicting the emergence and evolution of key terms over time. **(D)** Top 25 keywords exhibiting the strongest citation bursts. Red bars indicate years with significantly elevated citation counts for a given keyword, signaling emergent research trends that attracted intense scholarly attention.

Our study involves 148 research fields related to accidental falls in patients with PD (Figure 7A). Currently, research on PD tends to focuses on Neurosciences/Engineering, Biomedical/Engineering, Electrical & Electronic/Biochemistry & Molecular Biology/Chemistry, Physical/Pharmacology & Pharmacy/Engineering, and Multidisciplinary fields. It is evident that research directions regarding accidental falls in patients with PD are extensive, involving the fusion of multiple disciplines.

Typically, keywords represent the thematic focus and content of academic articles. By analyzing the keyword co-occurrence, we can rapidly gain insights into the research hotspots and prevailing trends within a particular field. We employed VOSviewer to construct a keyword co-occurrence network, which not only showcases frequently occurring keywords but also elucidates the co-occurrence relationships among keywords within the same article. We selected 193 keywords with a frequency of occurrence of ≥25 to construct a keyword co-occurrence network (Figure 7B) and listed the top 20 keywords based on their frequency. Apart from PD and fall, the keywords were mentioned over 150 times, accounting for 38% of the total link strength: gait (740), balance (565), elderly (529), risk factors (479), exercise (267), quality of life (267), walking (238), dementia (226), rehabilitation (217), postural sway (203), deep brain stimulation (201), performance (188), levodopa (191), motor (174), prevalence (166), multiple sclerosis (161), mobility (159), validation (156), and reliability (154). These keywords primarily revolve around the risk factors, clinical manifestations, and interventions for falls in patients with PD.

The timeline graph of keywords intuitively demonstrates their evolution process in the field and the research focus at each stage. Figure 7C shows nine active clusters of keywords. Progressive supranuclear palsy (#0) is the earliest and largest cluster. The earliest appearing keywords in this field include dementia, mortality, progression, impairment, and association. The latest research hotspots include survival, subtypes, epidemiology, and neurological rehabilitation. Machine learning (#8) is the newest emerging cluster, with keywords mainly related to activities of daily living, improvements made, accelerometer, wearable sensors, and deep learning. In general, research topics in this field have transitioned from early descriptive studies and mechanistic explorations of falls to comprehensive clinical assessments and subtype analyses, using new technologies such as artificial intelligence for assessment and monitoring to aid in the precise assessment of fall risk and to develop personalized preventive measures in an attempt to reduce falls in patients with PD and improve their quality of life.

Keywords exhibiting robust citation bursts can be employed as pivotal indicators of research hotspots and frontiers. As depicted in Figure 7D, the keyword with the highest burst intensity is progressive supranuclear palsy (strength 12.19), and that with the longest burst duration is cognitive function (2003–2013). Keywords currently experiencing bursts include rehabilitation, machine learning, validity, wearable sensors, epidemiology, and Parkinson’s disease. Hence, these topics are both current research hotspots and future research trends. By examining the citation burst situation of keywords, we can trace the development trajectory of the field, which typically began with the initial focus on the etiology and risk factor research, gradually transitioning to mechanistic exploration, and now concentrating on the rehabilitation measures for preventing falls in patients with PD and the application of new technologies to predict and prevent falls. Therefore, the current research hotspots and trends include the roles of rehabilitation exercise and tele-rehabilitation, artificial intelligence, and wearable devices in preventing falls in patients with PD.

## 4 Discussion

### 4.1 General information

Over a period of 20 years, 3,195 research articles related to accidental falls in patients with PD were published across 200 academic journals by 13,202 authors from 3,834 institutions in 87 countries. Since 2003, the annual publication volume in this field has seen a steady and rapid growth, with the number of publications in 2022 being 10 times that of 2003. This result indicates that accidental falls in patients with PD are one of the more popular research areas, attracting significant academic attention.

National and institutional visualization analyses show that the United States and the United Kingdom are the top-ranked countries in terms of *N_p_*, *N_c_*, and H-index. Five of these top 10 high-yield institutions are in the United States, and two are in the United Kingdom. The United States and the United Kingdom also have high betweenness centrality, indicating their crucial bridging role in the global cooperation network in this field. In terms of institutional collaboration, there is a close relationship between Tel Aviv University of Israel and Harvard University and Rush University of the United States. Hence, it can be suggested that the United States and the United Kingdom dominate the research in accidental falls in patients with PD. This dominance is likely related to the local governments’ emphasis and support policies for this field, as well as a higher degree of population aging in these countries. As the trend of population aging becomes more pronounced, the need to prevent and manage common complications in the older population, such as falls, will become increasingly urgent. Hence, investment in related research will continue to increase.

An analysis of journals and fields reveals that *Parkinsonism & Related Disorders* and *Movement Disorders* are at the forefront in terms of publication output and citation impact, underscoring the pivotal role that these two journals play in the research domain of accidental falls among individuals with PD (Supplementary Table S3; Figure 3). Notably, three of the top 10 high-yield journals belong to the Q1 category of the JCR, indicating that research endeavors within this field garner substantial recognition and significance within the global academic community. These popular journals mainly involve disciplines related to Medicine, Medical, and Clinical studies, consistent with the analysis of journal overlay maps. The current research primarily focuses on exploring the mechanisms and interventions for falls in patients with PD. At the same time, emerging trends in fall management strategies, the evaluation of intervention effects, and the application of new technologies have also been noted.

In terms of authors, Bloem, Bastiaan R from the Netherlands and Hausdorff, Jeffrey and Giladi, Nir from Israel have published the highest number of articles, with the highest citation counts and H-index. Bloem, Bastiaan R has the highest betweenness centrality, forming the largest cluster in both cooperation networks and co-citations, indicating his outstanding academic status in this field. Bloem, Bastiaan R and his team have explored various aspects of falls in patients with PD, including pathophysiology, the relationship between cognition and gait, the relationship between falls and FoG, physical therapy and exercise interventions, and comprehensive care.

According to Supplementary Table S5, the top 10 most-cited articles, with four articles being original research articles, primarily address the clinical characteristics of falls in patients with PD, physiological and psychological behavioral factors related to falls, risk prediction, and intervention measures (20–29). The highest betweenness centrality belongs to Nutt JG, who first proposed a close relationship between the FoG and falls in PD. Cluster dependency analysis indicated the following as the current research hotspots and future research trends: fear of falling, inertial sensors, exercise, and FoG. Citation burst analysis indicates research outcomes that have attracted widespread attention and frequent citations within a certain period, effectively capturing research dynamics and hotspot transitions in this field. The earliest citation burst began in 2001, with the strongest burst from a 2007 meta-analysis by Pickering RM et al., and a 2010 article by Kerr GK et al. attracted a sustained citation burst for 7 years. Among the five papers still experiencing citation bursts, three are articles and two are reviews. These papers comprehensively discuss the clinical diagnostic criteria for Parkinson’s motor disorders, gait impairment, fall subtypes in PD, along with the assessment and intervention of falls.

### 4.2 Hotspots and trends

One of the quintessential functions of bibliometrics analysis is its ability to process and analyze vast quantities of data, providing researchers with invaluable insights into the dynamic landscape of research trends (11). An investigation of frequently occurring keywords will help in the identifying shifting paradigms and prominent thematic areas, which are essential for understanding the evolutionary trajectory of a particular academic domain (11).

Before exploring specific analyses, let us first acquire a macro-level perspective on the evolution of keywords related to accidental falls among individuals with PD, spanning the period from 2003 to 2023. As shown in Figure 7C, the early stages of research primarily focused on the characteristics and risk factors of falls in PD, emphasizing the role of cognitive decline and basal ganglia lesions in falls. Subsequently, the focus shifted to exploring the pathophysiological mechanisms related to falls and attempting intervention methods. Through epidemiological surveys, longitudinal follow-ups, and randomized controlled trials, researchers analyzed the occurrence, development patterns, and influencing factors of falls in patients with PD. In recent years, research hotspots have mainly centered on rehabilitation measures as well as on the use of new technologies to predict and prevent falls. Keywords, such as rehabilitation, machine learning, validity, wearable sensors, and epidemiology, have been gaining prominence since 2019, reflecting the development trends in this field. Therefore, the roles of rehabilitation exercise and tele-rehabilitation, artificial intelligence, and wearable devices in preventing falls in patients with PD have been identified as the current research hotspots and trends.

#### 4.2.1 Rehabilitation exercise and tele-rehabilitation in preventing falls in patients with PD

Parkinson’s Disease is a progressively worsening neurodegenerative disorder, primarily characterized by motor dysfunction, cognitive impairment, and autonomic nervous system disturbances (30). As the disease progresses, patients often experience gait disturbances and decreased balance, significantly increasing the risk of falls (30). Falls not only cause a direct physical harm to patients but also exacerbate psychological burdens, increase medical costs, and reduce the quality of life (4, 5). Therefore, preventing and reducing falls in patients with PD has become a current research focus. Exercise training, a non-pharmacological intervention, has been proven to effectively improve both motor and non-motor symptoms in patients with PD by enhancing their muscle strength, improving posture control, increasing coordination, and boosting mental health. Consequently, the risk of fall significantly decreases and the quality of life increases for both patients and caregivers (6, 9, 31, 32). In recent years, various types of exercise training programs have been widely applied in the treatment of PD (32).

Home exercise games, protective step training, music therapy, Tai Chi and tele-rehabilitation are some of the methods that can improve balance and reduce fall risk in patients with PD (6, 31–33). A study involving 22 patients with PD undergoing a 4-week split-belt treadmill gait adaptation training found that they could effectively learn and retain the ability to overcome movement perturbations (10). Pelicioni et al. reported that a 12-week program combining home exercise games with unsupervised volitional step training improved the balance recovery of patients from induced perturbations, volitional stepping time, and stepping accuracy during cognitively challenging tests (34). Protective step training and music therapy improve balance through rhythmic stimulation (35, 36). Tai Chi not only enhances gait and stability but also aids in cognitive improvement, making it a beneficial exercise for reducing fall risk in this population (37, 38).

Tele-rehabilitation, as an adjunct, plays a crucial role in home exercise, fall reporting, and monitoring, thereby enhancing the health-related quality of life (HRQoL) of patients (39). Afshari et al. implemented video-based tele-physical/occupational therapy rehabilitation for 15 patients with PD and their caregivers and showed that fall risk decreased and home safety improved after 6 months (40). Li et al. developed a machine learning-based model to automatically recognize FOG in patients with PD using videos captured by smartphones. This model can be used for rapid assessment of FoG in the home environment as well as for remote management of FoG-PD (41). These applications did not increase the incidence of severe adverse events and were found to be simple, acceptable, and user-friendly for patients with PD (42, 43).

However, no standardized treatment protocol has yet been developed. In addition, the effectiveness of rehabilitation exercises may vary among individuals (32). Therefore, rehabilitation plans should be personalized based on the specific condition of each patient. Multidisciplinary team cooperation, including physical therapists and exercise trainers, is essential for developing and implementing effective rehabilitation exercise programs (6, 31). Future research should continue to explore the long-term efficacy and feasibility of different types of rehabilitation exercises in preventing falls in patients with PD to optimize intervention strategies and further benefit the patient population.

#### 4.2.2 Role of artificial intelligence in preventing falls in patients with PD

In recent years, machine learning has shown broad application prospects in health care. Its use in preventing falls among patients with PD has garnered increasing attention (44). Owing to its powerful data processing and analysis capabilities, machine learning offers new approaches for accurate and effective risk assessment, early prediction, intelligent monitoring, and rehabilitation exercises for patients with PD (44, 45).

For fall risk assessment, combining smartphone inertial sensors with actual measurements and analyzing fall-related risk factors through machine learning has led to the development of simple, fast and accurate fall risk assessment programs, providing a new decision-making model for fall screenings (46, 47). In early prediction, Juwara et al. developed a machine learning-based model to predict the loss of balance and falls in the mid-to-late stage in patients with PD, effectively predicting fall risk (48). Venuto et al. developed a dynamic progression clinical prediction model based on changes in motor disorder scores, which can predict walking ability in patients with PD (49). Additionally, machine learning plays a crucial role in intelligent monitoring, assisted decision-making, and personalized intervention for falls in patients with PD through data collection and analysis (50–52).

AI-driven virtual reality and gamified training systems provide patients with PD a safe and realistic environment. These systems can design customized comprehensive training programs for balance, gait and cognition based on individual patient conditions, enhancing their motor coordination abilities (53–55). A previous meta-analysis indicated that virtual reality (VR)-based balance training is more effective in immediately improving balance abilities in patients with PD post-training, especially in individuals with high baseline postural instability (55). Non-immersive VR can improve balance, reducing the risk and frequency of falls (53). However, challenges such as data privacy and security, model interpretability and credibility, and individual variability remain (45). Hence, future research should continue to address these issues to promote the widespread clinical application of machine learning, further improving the quality of life of patients with PD (45).

#### 4.2.3 Role of wearable devices in preventing falls in patients with PD

Wearable devices play a significant role in preventing falls in patients with PD by providing real-time monitoring, data analysis, and personalized prevention plans. These devices also assist with gait training and enhance the autonomy and compliance of exercises. Consequently, they themselves have become a research hotspot in recent years. Portable smart devices integrating motion sensors and physiological monitoring have shown broad application prospects in the health care, particularly in improving self-management for patients with chronic diseases (56–58). For patients with PD at a high risk of falls, wearable devices offer new solutions for fall prevention through convenient monitoring and data collection capabilities (59–61).

Recent research focuses on the relationship between gait abnormalities and fall risk in PD, with wearable devices providing new opportunities for fall prediction and continuous monitoring (58, 60). A previous meta-analysis indicated that using wearable devices to capture FOG and fall incidents in patients with PD can effectively prevent falls (62). Inertial sensor gait data have demonstrated that they are capable of providing personalized and accurate risk assessments (63). Wearable fall detection systems based on micro-electromechanical systems inertial sensors can distinguish between fall behaviors and normal activities by capturing gait and turning data, issuing warnings in advance (64, 65). Wearable devices with inertial measurement units record spatiotemporal gait characteristics in the daily lives of patients, providing references for fall risk assessment (66). Botonis et al. developed an anti-fall safety airbag vest with built-in wearable sensors to protect patients from accidental falls and reduce impact (67). Additionally, wearable devices equipped with voice recognition and video call functions can intelligently seek help in the event of a fall, accelerating rescue response and mitigating fall consequences (68).

Robot-assisted therapy not only improves HRQoL in patients with PD but also enhances cognition, psychological behavior and fear of falling (69). Wearable sensors are also used to assist with exercise, remote monitoring, and improving treatment compliance (69, 70). Kim et al. developed a soft wearable robot that enhances hip flexion to assist patients with PD in walking, enabling them to walk longer distances, at faster speeds and with improved gait quality (71). Adaptive robot-assisted ground gait training can improve the hip range of motion, gait speed, and stride length in patients with PD, reduce stride duration, and lower the fall risk (72). However, different patients have varying levels of acceptance and usage habits regarding wearable devices, which may affect their promotion and effectiveness of application. Hence, future research should continue to optimize device performance, address data privacy and security issues, and enhance patient acceptance and compliance to achieve widespread clinical application of wearable devices, further improving the quality of life of such patients.

### 4.3 Limitations

This study is subject to several limitations that warrant acknowledgment. Firstly, all data were sourced from WoSCC. Although this database includes most major publications, some relevant literature might still be missing. Secondly, the analysis only covered published literature, excluding ongoing research or unpublished data, which might introduce a publication bias. Lastly, the analytical software employed, such as CiteSpace and VOSviewer, inherently possesses certain limitations. For instance, terms extracted from titles, abstracts, and keywords may exhibit substantial variability during cluster analysis. In addition, there is no guarantee that all synonymous terms will be successfully grouped together.

## 5 Conclusion

The number of publications on accidental falls in patients with PD has been increasing annually, indicating a growing interest in this research area. The United States and the United Kingdom are the major contributors to this field. The journals *Parkinsonism & Related Disorders* and *Movement Disorders* demonstrate a significant influence in this domain. Professor Bloem Bastiaan R from the Netherlands has made outstanding contributions to this research area. Currently, Neurosciences and Biomedical are the most prominent research directions in this field. Rehabilitation exercises for preventing falls in patients with PD, as well as the application of machine learning and wearable technology, are current research hotspots and trends. Our study may help construct a knowledge map of the field, identify and analyze development trends and research hotspots, provide researchers with valuable references, and guide further in-depth studies in this field. Our bibliometric analysis provides an objective perspective on the research of accidental falls in patients with PD, helping scholars track knowledge development and identify future research directions.

## Data availability statement

Publicly available datasets were analyzed in this study. This data can be found here: https://www.webofscience.com/wos/woscc/summary/d8048105-c0b3-437e-846e-aae1b003a4a3-eb44760c/times-cited-descending/1.

## Author contributions

LS: Data curation, Funding acquisition, Software, Visualization, Writing – original draft, Writing – review & editing. BY: Supervision, Writing – original draft, Writing – review & editing.

## References

[1] Eckstrom E Vincenzo JL Casey CM Gray S Cosley K Caulley J . American Geriatrics Society response to the world falls guidelines. J Am Geriatr Soc. (2024) 72:1669–86. doi: 10.1111/jgs.18734, PMID: 38131656 PMC11187658

[2] Montero-Odasso M van der Velde N Martin FC Petrovic M Tan MP Ryg J . World guidelines for falls prevention and management for older adults: a global initiative. Age Ageing. (2022) 51:1–36. doi: 10.1093/ageing/afac205, PMID: 36178003 PMC9523684

[3] Pelicioni PHS Menant JC Latt MD Lord SR. Falls in Parkinson's disease subtypes: risk factors, locations and circumstances. Int J Environ Res Public Health. (2019) 16:1–9. doi: 10.3390/ijerph16122216, PMID: 31234571 PMC6616496

[4] Qamar MA Rota S Batzu L Subramanian I Falup-Pecurariu C Titova N . Chaudhuri's dashboard of vitals in Parkinson's syndrome: an unmet need underpinned by real life clinical tests. Front Neurol. (2023) 14:1174698. doi: 10.3389/fneur.2023.1174698, PMID: 37305739 PMC10248458

[5] Yilmaz FT Celik S Anataca G Sakar EM. Associations of nonmotor symptom burden, activities of daily living, and fear of falling in Parkinson disease. J Neurosci Nurs. (2023) 55:137–42. doi: 10.1097/jnn.0000000000000712 37348005

[6] Camicioli R Morris ME Pieruccini-Faria F Montero-Odasso M Son S Buzaglo D . Prevention of falls in Parkinson's disease: guidelines and gaps. Mov Disord Clin Pract. (2023) 10:1459–69. doi: 10.1002/mdc3.13860, PMID: 37868930 PMC10585979

[7] Lin M Mi TM Jia Q Han C Chhetri JK Chan P. Gait variability is sensitive to detect Parkinson's disease patients at high fall risk. Int J Neurosci. (2022) 132:888–93. doi: 10.1080/00207454.2020.1849189, PMID: 33256488

[8] Meira H Rosa I Valenca G Oliveira-Filho J Almeida L. Predictors of falls with injuries in people with Parkinson's disease. Mov Disord. (2020) 35:S535. doi: 10.1002/mdc3.13636 PMC9941941 36825046

[9] Feng FF Xu HC Sun Y Zhang X Li N Sun X . Exercise for prevention of falls and fall-related injuries in neurodegenerative diseases and aging-related risk conditions: a meta-analysis. Front Endocrinol. (2023) 14:1187325. doi: 10.3389/fendo.2023.1187325, PMID: 37534209 PMC10393124

[10] Hulzinga F Seuthe J D'Cruz N Ginis P Nieuwboer A Schlenstedt C. Split-Belt treadmill training to improve gait adaptation in Parkinson's disease. Mov Disord. (2023) 38:92–103. doi: 10.1002/mds.29238, PMID: 36239376

[11] Donthu N Kumar S Mukherjee D Pandey N Lim WM. How to conduct a bibliometric analysis: An overview and guidelines. J Bus Res. (2021) 133:285–96. doi: 10.1016/j.jbusres.2021.04.070

[12] Moral-Muñoz JA Herrera-Viedma E Santisteban-Espejo A Cobo MJ. Software tools for conducting bibliometric analysis in science: an up-to-date review. Profes Inform. (2020) 29:1–20. doi: 10.3145/epi.2020.ene.03

[13] Qu FL Wang GW Wen P Liu XY Zeng XH. Knowledge mapping of immunotherapy for breast cancer: a bibliometric analysis from 2013 to 2022. Hum Vaccin Immunother. (2024) 20:1–3. doi: 10.1080/21645515.2024.2335728, PMID: 38563136 PMC10989689

[14] Song YP Liu JL Zong CZ Zhang FS Ren YF Ching YL . A bibliometric study on trends in chiropractic research from 1920 to 2023. Complement Ther Med. (2024) 82:103038. doi: 10.1016/j.ctim.2024.103038, PMID: 38582375

[15] Xu ZH Guan C Cheng ZJ Zhou HL Qin WT Wan M . Research trends and hotspots of circular RNA in cardiovascular disease: a bibliometric analysis. Non-Coding RNA Res. (2024) 9:930–44. doi: 10.1016/j.ncrna.2024.04.002, PMID: 38680417 PMC11047193

[16] Yuan GW Yang YQ Lin YJ Lin JR Wu YC. Current status and development trends in CKD with frailty research from 2000 to 2021: a bibliometric analysis. Ren Fail. (2024) 46:1–16. doi: 10.1080/0886022x.2023.2292142, PMID: 38178378 PMC10773684

[17] Zhu J Liu W. A tale of two databases: the use of Web of Science and Scopus in academic papers. Scientometrics. (2020) 123:321–335. doi: 10.1007/s11192-020-03387-8

[18] van Eck NJ Waltman L. Software survey: VOSviewer, a computer program for bibliometric mapping. Scientometrics. (2010) 84:523–38. doi: 10.1007/s11192-009-0146-3, PMID: 20585380 PMC2883932

[19] Chen CM . CiteSpace II: detecting and visualizing emerging trends and transient patterns in scientific literature. J Am Soc Inf Sci Technol. (2006) 57:359–77. doi: 10.1002/asi.20317

[20] Bloem BR Hausdorff JA Visser JE Giladi N. Falls and freezing of gait in Parkinson’s disease: a review of two interconnected, episodic phenomena. Mov Disord. (2004) 19:871–84. doi: 10.1002/mds.20115, PMID: 15300651

[21] Braak H Rüb U Gai WP Del Tredici K. Idiopathic Parkinson’s disease:: possible routes by which vulnerable neuronal types may be subject to neuroinvasion by an unknown pathogen. J Neural Transm. (2003) 110:517–36. doi: 10.1007/s00702-002-0808-2 12721813

[22] Gallagher P Ryan C Byrne S Kennedy J O’Mahony D. STOPP (screening tool of older Person’s prescriptions) and START (screening tool to alert doctors to right treatment). Consensus validation. Int J Clin Pharmacol Ther. (2008) 46:72–83. doi: 10.5414/CPP46072, PMID: 18218287

[23] Gerfen CR Surmeier DJ. Modulation of striatal projection systems by dopamine In: Hyman SE Jessell TM Shatz CJ Stevens CF Zoghbi HY, editors. Annual Review of Neuroscience, vol. 34 (2011). 441–66. 10.1146/annurev-neuro-061010-113641 PMC3487690 21469956

[24] Healy DG Falchi M O’Sullivan SS Bonifati V Durr A Bressman S . Phenotype, genotype, and worldwide genetic penetrance of LRRK2-associated Parkinson’s disease: a case-control study. Lancet Neurol. (2008) 7:583–90. doi: 10.1016/s1474-4422(08)70117-0, PMID: 18539534 PMC2832754

[25] Hely MA Morris JGL Reid WGJ Trafficante R. Sydney multicenter study of Parkinson’s disease: non-L-dopa-responsive problems dominate at 15 years. Mov Disord. (2005) 20:190–9. doi: 10.1002/mds.20324, PMID: 15551331

[26] Hely MA Reid WGJ Adena MA Halliday GA Morris JGL. The Sydney multicenter study of Parkinson’s disease: the inevitability of dementia at 20 years. Mov Disord. (2008) 23:837–44. doi: 10.1002/mds.21956, PMID: 18307261

[27] Montero-Odasso M Verghese J Beauchet O Hausdorff JM. Gait and cognition: a complementary approach to understanding brain function and the risk of falling. J Am Geriatr Soc. (2012) 60:2127–36. doi: 10.1111/j.1532-5415.2012.04209.x, PMID: 23110433 PMC3498517

[28] Olanow CW Stern MB Sethi K. The scientific and clinical basis for the treatment of Parkinson disease (2009). Neurology. (2009) 72:S1–S136. doi: 10.1212/WNL.0b013e3181a1d44c, PMID: 19470958

[29] Yogev-Seligmann G Hausdorff JM Giladi N. The role of executive function and attention in gait. Mov Disord. (2008) 23:329–42. doi: 10.1002/mds.21720, PMID: 18058946 PMC2535903

[30] Bloem BR Okun MS Klein C. Parkinson's disease. Lancet. (2021) 397:2284–303. doi: 10.1016/s0140-6736(21)00218-x 33848468

[31] Allen NE Canning CG Almeida LRS Bloem BR Keus SH Löfgren N . Interventions for preventing falls in Parkinson's disease. Cochrane Database Syst Rev. (2022) 2022:CD011574. doi: 10.1002/14651858.CD011574.pub2, PMID: 35665915 PMC9169540

[32] Ernst M Folkerts AK Gollan R Lieker E Caro-Valenzuela J Adams A . Physical exercise for people with Parkinson’s disease: a systematic review and network meta-analysis. Cochrane Database Syst Rev. (2024) 2024:CD013856. doi: 10.1002/14651858.CD013856.pub3, PMID: 38588457 PMC11001292

[33] Zhou Z Zhou R Wei W Luan R Li K. Effects of music-based movement therapy motor function, balance, gait, mental health, and quality of life for patients with Parkinson’s disease: a systematic review and meta-analysis. Clin. Rehabil. (2021) 35:937–951., PMID: 33517767 10.1177/0269215521990526

[34] Pelicioni PHS Lord SR Menant JC Chaplin C Canning C Brodie MA . Combined reactive and volitional step training improves balance recovery and stepping reaction time in people with Parkinson's disease: a randomised controlled trial. Neurorehabil Neural Repair. (2023) 37:694–704. doi: 10.1177/15459683231206743, PMID: 37864439 PMC10666522

[35] De Cock VC Dotov D Damm L Lacombe S Ihalainen P Picot MC . BeatWalk: personalized music-based gait rehabilitation in Parkinson's disease. Front Psychol. (2021) 12:655121. doi: 10.3389/fpsyg.2021.655121, PMID: 33981279 PMC8109247

[36] Lockhart T Frames C Olson M Moon SH Peterson D Lieberman A. Effects of protective step training on proactive and reactive motor adaptations in Parkinson's disease patients. Front Neurol. (2023) 14:1211441. doi: 10.3389/fneur.2023.1211441, PMID: 37965161 PMC10642212

[37] Li G Huang P Cui SS He YC Jiang QY Li BY . Tai chi improves non-motor symptoms of Parkinson's disease: one-year randomized controlled study with the investigation of mechanisms. Parkinsonism Relat Disord. (2024a) 120:105978. doi: 10.1016/j.parkreldis.2023.105978, PMID: 38244460

[38] Toloraia K Gschwandtner U Fuhr P. High-frequency multimodal training with a focus on tai chi in people with Parkinson's disease: a pilot study. Front Aging Neurosci. (2024) 16:1335951. doi: 10.3389/fnagi.2024.1335951, PMID: 38425785 PMC10902121

[39] Salchow-Hoemmen C Skrobot M Jochner MCE Schauer T Kuehn AA Wenger N. Review-emerging portable technologies for gait analysis in neurological disorders. Front Hum Neurosci. (2022) 16:768575. doi: 10.3389/fnhum.2022.768575, PMID: 35185496 PMC8850274

[40] Afshari M Hernandez AV Joyce JM Hauptschein AW Trenkle KL Stebbins GT . A novel home-based telerehabilitation program targeting fall prevention in Parkinson disease: a preliminary trial. Neurol Clin Pract. (2024) 14:e200246. doi: 10.1212/CPJ.0000000000200246 38213401 PMC10781563

[41] Li WD Chen XJ Zhang JT Lu JJ Zhang CC Bai HM . Recognition of freezing of gait in Parkinson's disease based on machine vision. Front Aging Neurosci. (2022) 14:921081. doi: 10.3389/fnagi.2022.921081 35912091 PMC9329960

[42] Lee BC An J Kim J Lai EC. Performing dynamic weight-shifting balance exercises with a smartphone-based wearable telerehabilitation system for home use by individuals with Parkinson's disease: a proof-of-concept study. IEEE Trans Neural Syst Rehabil Eng. (2023) 31:456–63. doi: 10.1109/tnsre.2022.3226368, PMID: 36455080 PMC10079610

[43] Ye B How TV Chu CH Mihailidis A. Dementia care apps for people with dementia and informal caregivers: a systematic review protocol. Gerontology. (2021) 67:633–638. doi: 10.1159/000514838, PMID: 33774646 PMC8619761

[44] Bargiotas I Wang DP Mantilla J Quijoux F Moreau A Vidal C . Preventing falls: the use of machine learning for the prediction of future falls in individuals without history of fall. J Neurol. (2023) 270:618–31. doi: 10.1007/s00415-022-11251-3, PMID: 35817988 PMC9886639

[45] Wu P Cao BW Liang ZD Wu M. The advantages of artificial intelligence-based gait assessment in detecting, predicting, and managing Parkinson's disease. Front Aging Neurosci. (2023) 15:1191378. doi: 10.3389/fnagi.2023.1191378, PMID: 37502426 PMC10368956

[46] Fong KNK Chung RCK Sze PPC Ng CKM. Factors associated with fall risk of community-dwelling older people: a decision tree analysis. Digital Health. (2023) 9:205520762311812. doi: 10.1177/20552076231181202, PMID: 37325076 PMC10262624

[47] McManus K Greene BR Ader LGM Caulfield B. Development of data-driven metrics for balance impairment and fall risk assessment in older adults. IEEE Trans. Biomed. Eng. (2022) 69:2324–2332. doi: 10.1109/TBME.2022.3142617 35025734

[48] Juwara L Cressatti M Galindez JM Drammeh PS Velly AM Schipper HM. Development and internal validation of a prognostic model for loss of balance and falls in mid-to late-stage Parkinson's disease. Neurol Sci. (2024) 45:2027–33. doi: 10.1007/s10072-023-07220-x, PMID: 38060035

[49] Venuto CS Smith G Herbst K Zielinski R Yung NCW Grosset DG . Predicting ambulatory capacity in Parkinson's disease to analyze progression, biomarkers, and trial design. Mov Disord. (2023) 38:1774–85. doi: 10.1002/mds.29519, PMID: 37363815 PMC10615710

[50] Bajpai R Khare S Joshi D. A multimodal model-fusion approach for improved prediction of freezing of gait in Parkinson's disease. IEEE Sensors J. (2023) 23:16168–75. doi: 10.1109/jsen.2023.3284656

[51] Shah VV Jagodinsky A McNames J Carlson-Kuhta P Nutt JG El-Gohary M . Gait and turning characteristics from daily life increase ability to predict future falls in people with Parkinson's disease. Front Neurol. (2023a) 14:1096401. doi: 10.3389/fneur.2023.1096401, PMID: 36937534 PMC10015637

[52] Yang PK Filtjens B Ginis P Goris M Nieuwboer A Gilat M . Freezing of gait assessment with inertial measurement units and deep learning: effect of tasks, medication states, and stops. J Neuroeng Rehabil. (2024) 21:24. doi: 10.1186/s12984-024-01320-1, PMID: 38350964 PMC10865632

[53] Garcia-Lopez H Obrero-Gaitan E Castro-Sanchez AM Lara-Palomo IC Nieto-Escamez FA Cortes-Perez I. Non-immersive virtual reality to improve balance and reduce risk of falls in people diagnosed with Parkinson's disease: a systematic review. Brain Sci. (2021) 11:1–14. doi: 10.3390/brainsci11111435, PMID: 34827433 PMC8615507

[54] Pezzi L Di Matteo A Insabella R Mastrogiacomo S Baldari C Reiss VM . How cognitive reserve should influence rehabilitation choices using virtual reality in Parkinson's disease. Parkinsons Dis. (2022) 2022:1–17. doi: 10.1155/2022/7389658, PMID: 36160828 PMC9507627

[55] Sarasso E Gardoni A Tettamanti A Agosta F Filippi M Corbetta D. Virtual reality balance training to improve balance and mobility in Parkinson's disease: a systematic review and meta-analysis. J Neurol. (2022) 269:1873–88. doi: 10.1007/s00415-021-10857-3, PMID: 34713324

[56] Ates HC Nguyen PQ Gonzalez-Macia L Morales-Narvaez E Guder F Collins JJ . End-to-end design of wearable sensors. Nat Rev Mater. (2022) 7:887–907. doi: 10.1038/s41578-022-00460-x, PMID: 35910814 PMC9306444

[57] Jadhwani PL Harjpal P. A review of artificial intelligence-based gait evaluation and rehabilitation in Parkinson's disease. Cureus. (2023) 15:e47118. doi: 10.7759/cureus.47118, PMID: 38021909 PMC10648061

[58] Salaorni F Bonardi G Schena F Tinazzi M Gandolfi M. Wearable devices for gait and posture monitoring via telemedicine in people with movement disorders and multiple sclerosis: a systematic review. Expert Rev Med Dev. (2024) 21:121–40. doi: 10.1080/17434440.2023.2298342, PMID: 38124300

[59] Dasgupta P VanSwearingen J Godfrey A Redfern M Montero-Odasso M Sejdic E. Acceleration gait measures as proxies for motor skill of walking: a narrative review. IEEE Trans Neural Syst Rehabil Eng. (2021) 29:249–61. doi: 10.1109/tnsre.2020.3044260, PMID: 33315570 PMC7995554

[60] Olson MC Lockhart TE. Predicting fall risk through automatic wearable monitoring: a systematic review. Int J Prognost Health Manag. (2021) 12:1–15. doi: 10.36001/IJPHM.2021.v12i4.2958

[61] Picerno P Iosa M D'Souza C Benedetti MG Paolucci S Morone G. Wearable inertial sensors for human movement analysis: a five-year update. Expert Rev Med Dev. (2021) 18:79–94. doi: 10.1080/17434440.2021.1988849, PMID: 34601995

[62] Huang TH Li M Huang JW. Recent trends in wearable device used to detect freezing of gait and falls in people with Parkinson's disease: a systematic review. Front Aging Neurosci. (2023) 15:1119956. doi: 10.3389/fnagi.2023.1119956, PMID: 36875701 PMC9975590

[63] Ullrich M Roth N Kuderle A Richer R Gladow T Gasner H . Fall risk prediction in Parkinson's disease using real-world inertial sensor gait data. IEEE J Biomed Health Inform. (2023) 27:319–28. doi: 10.1109/jbhi.2022.3215921, PMID: 36260566

[64] Ren K Chen ZL Ling Y Zhao J. Recognition of freezing of gait in Parkinson's disease based on combined wearable sensors. BMC Neurol. (2022) 22:229. doi: 10.1186/s12883-022-02732-z, PMID: 35729546 PMC9210754

[65] Xu Z Luo Y. A wearable Micro-electromechanical system inertial sensor system for fall behaviour detection based on a multi-level threshold algorithm. ECS J Solid State Sci Technol. (2023) 12:057013. doi: 10.1149/2162-8777/acd65f

[66] Moore J Stuart S McMeekin P Walker R Celik Y Pointon M . Enhancing free-living fall risk assessment: contextualizing mobility based IMU data. Sensors. (2023) 23:1–16. doi: 10.3390/s23020891, PMID: 36679685 PMC9866998

[67] Botonis OK Harari Y Embry KR Mummidisetty CK Riopelle D Giffhorn M . Wearable airbag technology and machine learned models to mitigate falls after stroke. J Neuroeng Rehabil. (2022) 19:60. doi: 10.1186/s12984-022-01040-4, PMID: 35715823 PMC9205156

[68] Shah VV McNames J Carlson-Kuhta P Nutt JG El-Gohary M Sowalsky K . Effect of levodopa and environmental setting on gait and turning digital markers related to falls in people with Parkinson's disease. Mov Disord Clin Pract. (2023b) 10:223–30. doi: 10.1002/mdc3.13601, PMID: 36825056 PMC9941945

[69] Zanatta F Farhane-Medina NZ Adorni R Steca P Giardini A D'Addario M . Combining robot-assisted therapy with virtual reality or using it alone? A systematic review on health-related quality of life in neurological patients. Health Qual Life Outcomes. (2023) 21:18. doi: 10.1186/s12955-023-02097-y, PMID: 36810124 PMC9942343

[70] Denk D Herman T Zoetewei D Ginis P Brozgol M Thumm PC . Daily-living freezing of gait as quantified using wearables in people with Parkinson disease: comparison with self-report and provocation tests. Phys Ther. (2022) 102:1–11. doi: 10.1093/ptj/pzac129, PMID: 36179090 PMC10071496

[71] Kim J Porciuncula F Yang HD Wendel N Baker T Chin A . Soft robotic apparel to avert freezing of gait in Parkinson's disease. Nat Med. (2024) 30:177–85. doi: 10.1038/s41591-023-02731-8, PMID: 38182783

[72] Otlet V Vandamme C Warlop T Crevecoeur F Ronsse R. Effects of overground gait training assisted by a wearable exoskeleton in patients with Parkinson's disease. J Neuroeng Rehabil. (2023) 20:156. doi: 10.1186/s12984-023-01280-y, PMID: 37974229 PMC10655429

