# Supplementary material for: Knowledge mapping and research trends of accidental falls in patients with Parkinson’s disease from 2003 to 2023: a bibliometric analysis
Source: Front Neurol. 2024 Aug 22;15:1443799. doi: 10.3389/fneur.2024.1443799 (PMC11375799; doi:10.3389/fneur.2024.1443799)
Supplement: Supplementary file 1 [file Table_1.docx]

Table S1. The Top10 countries in the research of accidental falls in patients with Parkinson Disease.

| **Rank** | **Countries** | **Np** | **Countries** | **Nc** | **Countries** | **H-Index** | **Countries** | **Total Link Strength** |
| --- | --- | --- | --- | --- | --- | --- | --- | --- |
| 1 | USA | 1117 | USA | 53611 | USA | 111 | USA | 743 |
| 2 | England | 377 | England | 20064 | England | 70 | England | 577 |
| 3 | China | 282 | Australia | 16949 | Netherlands | 62 | Netherlands | 388 |
| 4 | Italy | 263 | Israel | 14921 | Israel | 60 | Germany | 380 |
| 5 | Germany | 252 | Netherlands | 13673 | Australia | 59 | Italy | 350 |
| 6 | Australia | 233 | Canada | 11440 | Germany | 54 | Canada | 316 |
| 7 | Canada | 231 | Germany | 11087 | Italy | 54 | France | 278 |
| 8 | Netherlands | 191 | Italy | 10364 | Canada | 52 | Australia | 268 |
| 9 | France | 157 | France | 7430 | France | 43 | Israel | 236 |
| 10 | Israel | 146 | China | 5908 | China | 39 | Spain | 216 |
