# Supplementary material for: Knowledge mapping and research trends of accidental falls in patients with Parkinson’s disease from 2003 to 2023: a bibliometric analysis
Source: Front Neurol. 2024 Aug 22;15:1443799. doi: 10.3389/fneur.2024.1443799 (PMC11375799; doi:10.3389/fneur.2024.1443799)
Supplement: Supplementary file 2 [file Table_2.docx]

Table S2. The top 10 productive research institutions with publications concerning accidental falls in patients with Parkinson Disease.

| **Rank** | **Institution** | **Np** | **Country** | **Institution** | **Nc** | **Country** | **Institution** | **H-Index** | **Country** |
| --- | --- | --- | --- | --- | --- | --- | --- | --- | --- |
| 1 | Tel Aviv University | 119 | Israel | Harvard University | 14596 | USA | Harvard University | 57 | USA |
| 2 | University Of London | 115 | England | Tel Aviv University | 13513 | Israel | Tel Aviv University | 56 | Israel |
| 3 | Radboud University Nijmegen | 110 | Netherlands | University Of London | 9017 | England | Radboud University Nijmegen | 48 | Netherlands |
| 4 | Harvard University | 102 | USA | Radboud University Nijmegen | 8065 | Netherlands | University Of London | 43 | England |
| 5 | US Department Of Veterans Affairs | 87 | USA | University Of Sydney | 6172 | Australia | US Department Of Veterans Affairs | 35 | USA |
| 6 | State University System Of Florida | 80 | USA | US Department Of Veterans Affairs | 4403 | USA | Newcastle University Uk | 35 | England |
| 7 | University Of California System | 80 | USA | Oregon Health Science University | 4220 | USA | University Of California System | 32 | USA |
| 8 | Newcastle University Uk | 77 | England | University Of Toronto | 3982 | Canada | Oregon Health Science University | 32 | USA |
| 9 | Oregon Health Science University | 77 | USA | Newcastle University Uk | 3727 | UK | Ku Leuven | 32 | Belgium |
| 10 | University Of Sydney | 72 | Australia | Ku Leuven | 3656 | Belgium | State University System Of Florida | 31 | USA |
