# Supplementary material for: Knowledge mapping and research trends of accidental falls in patients with Parkinson’s disease from 2003 to 2023: a bibliometric analysis
Source: Front Neurol. 2024 Aug 22;15:1443799. doi: 10.3389/fneur.2024.1443799 (PMC11375799; doi:10.3389/fneur.2024.1443799)
Supplement: Supplementary file 3 [file Table_3.docx]

Table S3. The top 10 productive academic journals with publications concerning accidental falls in patients with Parkinson Disease.

| **Rank** | **Journal** | **Np** | **%of3195** | **IF（JCR2022)** | **JCR Quatile** | **Journal** | **Nc** | **Journal** | **H-Index** |
| --- | --- | --- | --- | --- | --- | --- | --- | --- | --- |
| 1 | Parkinsonism & Related Disorders | 143 | 4.48% | 4.1 | Q2 | Movement Disorders | 15766 | Movement Disorders | 64 |
| 2 | Movement Disorders | 138 | 4.32% | 8.6 | Q1 | Parkinsonism & Related Disorders | 5313 | Parkinsonism & Related Disorders | 43 |
| 3 | Gait & Posture | 93 | 2.91% | 2.4 | Q4 | Neurology | 4182 | Gait & Posture | 30 |
| 4 | Frontiers In Neurology | 81 | 2.54% | 3.4 | Q2 | Brain | 4063 | Plos One | 27 |
| 5 | Plos One | 66 | 2.07% | 3.7 | Q2 | Journal Of Neurology Neurosurgery And Psychiatry | 2940 | Journal Of Neurology | 27 |
| 6 | Journal Of Parkinsons Disease | 65 | 2.03% | 5.2 | Q1 | Gait & Posture | 2834 | Journal Of Parkinsons Disease | 25 |
| 7 | Journal Of Neurology | 63 | 1.97% | 6.0 | Q1 | Journal Of Neurology | 2194 | Neurology | 25 |
| 8 | Sensors | 48 | 1.50% | 3.9 | Q2 | Plos One | 2025 | Journal Of Neurology Neurosurgery And Psychiatry | 24 |
| 9 | Parkinsons Disease | 43 | 1.35% | 3.2 | Q2 | Parkinsons Disease | 1760 | Brain | 23 |
| 10 | Bmc Neurology | 40 | 1.25% | 2.6 | Q3 | Physical Therapy | 1687 | Frontiers In Neurology | 20 |
