# Supplementary material for: Knowledge mapping and research trends of accidental falls in patients with Parkinson’s disease from 2003 to 2023: a bibliometric analysis
Source: Front Neurol. 2024 Aug 22;15:1443799. doi: 10.3389/fneur.2024.1443799 (PMC11375799; doi:10.3389/fneur.2024.1443799)
Supplement: Supplementary file 4 [file Table_4.docx]

Table S4. The top 10 productive authors with publications, H-Index and citation frequency concerning accidental falls in patients with Parkinson Disease.

| **Rank** | **Author** | **Np** | **Country** | **Author** | **H-Index** | **Country** | **Author** | **Nc** | **Country** |
| --- | --- | --- | --- | --- | --- | --- | --- | --- | --- |
| 1 | Bloem, Bastiaan | 93 | Netherlands | Hausdorff, Jeffrey | 51 | Israel | Hausdorff, Jeffrey | 12021 | Israel |
| 2 | Hausdorff, Jeffrey | 86 | Israel | Giladi, Nir | 47 | Israel | Giladi, Nir | 9983 | Israel |
| 3 | Giladi, Nir | 63 | Israel | Bloem, Bastiaan | 46 | Netherlands | Bloem, Bastiaan | 7477 | Netherlands |
| 4 | Rochester, Lynn | 57 | England | Rochester, Lynn | 29 | England | Rochester, Lynn | 3569 | England |
| 5 | Nieuwboer, Alice M | 45 | Belgium | Nieuwboer, Alice M | 28 | Belgium | Nieuwboer, Alice M | 3246 | Belgium |
| 6 | Horak, Fay B | 41 | USA | Horak, Fay B | 23 | USA | Herman, Talia | 2973 | Israel |
| 7 | Lord, Stephen R | 35 | Australia | Lord, Stephen R | 23 | Australia | Horak, Fay B | 2260 | USA |
| 8 | Canning, Colleen G | 31 | Australia | Canning, Colleen G | 23 | Australia | Canning, Colleen G | 2044 | Australia |
| 9 | Martin, Caryn | 30 | USA | Herman, Talia | 23 | Israel | Lord, Stephen R | 1937 | Australia |
| 10 | Earhart, Gammon M | 28 | USA | Mirelman, Anat | 21 | USA | Earhart, Gammon M | 1662 | USA |
