# Supplementary material for: Knowledge mapping and research trends of accidental falls in patients with Parkinson’s disease from 2003 to 2023: a bibliometric analysis
Source: Front Neurol. 2024 Aug 22;15:1443799. doi: 10.3389/fneur.2024.1443799 (PMC11375799; doi:10.3389/fneur.2024.1443799)
Supplement: Supplementary file 5 [file Table_5.docx]

Table S5. The top 10 co-cited reference concerning accidental falls in patients with Parkinson Disease.

| **Rank** | **Title** | **First Author** | **Journal** | **Year** | **Nc** | **Document Type** | **DOI** |
| --- | --- | --- | --- | --- | --- | --- | --- |
| 1 | The Sydney multicenter study of Parkinson's disease : The inevitability of dementia at 20 years | Hely, Mariese A. | Movement Disorders | 2008 | 1519 | Article | 10.1002/mds.21956 |
| 2 | The role of executive function and attention in gait | Yogev-Seligmann, Galit | Movement Disorders | 2008 | 1385 | Review | 10.1002/mds.21720 |
| 3 | Phenotype, genotype, and worldwide genetic penetrance of LRRK2-associated Parkinson's disease: a case-control study | Healy, Daniel G. | Lancet Neurology | 2008 | 1112 | Article | 10.1016/S1474-4422(08)70117-0 |
| 4 | Modulation of Striatal Projection Systems by Dopamine | Gerfen, Charles R. | Annual Review Of Neuroscience | 2011 | 1110 | Review | 10.1146/annurev-neuro-061010-113641 |
| 5 | Idiopathic Parkinson's disease:: possible routes by which vulnerable neuronal types may be subject to neuroinvasion by an unknown pathogen | Braak, H | Journal Of Neural Transmission | 2003 | 1103 | Review | 10.1007/s00702-002-0808-2 |
| 6 | STOPP (Screening Tool of Older Person's Prescriptions) and START (Screening Tool to Alert Doctors to Right Treatment). Consensus validation | Gallagher, P. | International Journal Of Clinical Pharmacology And Therapeutics | 2008 | 1006 | Review | 10.1093/ageing/afn197 |
| 7 | Falls and freezing of gait in Parkinson's disease: A review of two interconnected, episodic phenomena | Bloem, BR | Movement Disorders | 2004 | 901 | Review | 10.1002/mds.20115 |
| 8 | Sydney multicenter study of Parkinson's disease: non-L-dopa-responsive problems dominate at 15 years | Hely, MA | Movement Disorders | 2005 | 819 | Article | 10.1002/mds.20324 |
| 9 | Gait and Cognition: A Complementary Approach to Understanding Brain Function and the Risk of Falling | Montero-Odasso, Manuel | Journal Of The American Geriatrics Society | 2012 | 616 | Article | 10.1111/j.1532-5415.2012.04209.x |
| 10 | The scientific and clinical basis for the treatment of Parkinson disease (2009) | Olanow, C. Warren | Neurology | 2009 | 608 | Review | 10.1212/WNL.0b013e3181a1d44c |
